# Factors influencing maternal healthcare utilization in Papua New Guinea: Andersen’s behaviour model

**DOI:** 10.1186/s12905-023-02709-1

**Published:** 2023-10-21

**Authors:** Yan Li, Hao Li, Yi Jiang

**Affiliations:** 1 School of Public Health, Chongqing Medical University, Chongqing, China; 2 Second Affiliated Hospital of Soochow University, Jiangsu, China

**Keywords:** Maternal healthcare services, Antenatal care, Skilled birth attendant, Postnatal care, Papua New Guinea, Andersen’s behaviour model

## Abstract

**Background:**

Papua New Guinea (PNG) has recorded the highest maternal mortality ratio in the Western Pacific Region and faces major challenges in achieving SDG 3. Antenatal care (ANC), skilled birth attendant (SBA) and postnatal care (PNC) services are critical components of maternal healthcare services (MHS) for reducing maternal mortality and promoting maternal health in PNG. The study sought to assess the prevalence and determinants of ANC, SBA and PNC services amongst women in PNG.

**Methods:**

The study was conducted using the 2016–2018 Papua New Guinea Demographic and Health Survey. A total of 5248 reproductive-age women were considered as the analytical sample. The outcome variables were utilisation of ANC, SBA and PNC services. Chi-square test, multivariable logistic regression and dominance analysis were conducted. Statistical significance was set at *p* < 0.05.

**Results:**

The prevalence rates of ANC, SBA and PNC services were 52.3%, 58.7% and 26.6%, respectively. Women’s employment, education, media exposure, distance to health facility, household wealth, region, residence and parity were determinants of MHS utilisation. ANC, SBA and PNC services utilisation were all primarily influenced by enabling factors, followed by predisposing and need factors.

**Conclusions:**

This study demonstrated that enabling factors such as media exposure, distance to health facility, household wealth, region and residence have the greatest impact on MHS utilisation, followed by predisposing (working, education) and need factors (parity). Therefore, enabling factors should be prioritised when developing maternal health programmes and policies. For example, transport and health infrastructure should be strengthened and women’s education and vocational training should be increased, especially in Highlands region, Momase region and rural areas, to increase the utilisation of MHS.

## Introduction

Women’s survival, health and well-being are essential to eradicating extreme poverty, promoting development and resilience and achieving the Sustainable Development Goals (SDGs) [1]. Between 1990 and 2017, the global maternal mortality ratio fell from 385 to 211 per 100,000 live births, and significant progress has been made in controlling maternal mortality [2]. Every day, approximately 830 women worldwide die from preventable causes related to pregnancy or childbirth, most of which occur in low- and middle-income countries (LMICs) [3]. Papua New Guinea (PNG) has recorded the highest maternal mortality ratio (145 per 100,000 live births, 2017) in the Western Pacific Region [4] and faces major challenges in achieving SDG 3 [5]. The leading causes of maternal death include obstetric haemorrhage, sepsis, embolism, eclampsia and unsafe abortion, some of which can be effectively prevented and controlled through maternal healthcare [6].

Antenatal care (ANC), skilled birth attendant (SBA) and postnatal care (PNC) services have been proven to be necessary interventions to ensure maternal health [7] and are crucial to reducing maternal mortality and promoting maternal health and well-being in PNG. Although several empirical studies have been conducted on the issue of maternal healthcare services (MHS) utilisation in PNG [8, 9], they mainly focused on assessing factors associated with ANC or SBA services. In PNG, a comprehensive analysis of all three MHS is currently lacking.

In this study, Andersen’s behaviour model was adopted to analyse the factors associated with ANC, SBA and PNC services utilisation amongst women in PNG. Andersen’s behavioral model is a widely used classic model in the field of health service utilization research. In Andersen’s behavioral model, health service utilization is determined jointly by demand and supply factors. This model views individuals’ utilization behavior of health services as a decision-making process, which is influenced by a range of factors [10]. The use of Andersen’s behavioral model in this study helps us to gain a more comprehensive and in-depth understanding of the factors influencing PNG pregnant women’s use of medical and health services, thereby providing scientific evidence for improving PNG’s health policies and enhancing the utilization level of maternal health services. The model explains the influencing factors of individual access to healthcare services from three dimensions: predisposing, enabling and need factors [11]. Predisposing factors are socio-demographics characteristics; enabling factors are the conditions and resources to use healthcare services such as income, health insurance, health personnel and facilities density, price of services and region; and need factors are the most direct reasons to use healthcare services, including the perception of illness by the individual or his family and clinical evaluation [12].

## Materials and methods

### Data source

The data for this study were obtained from The Demographic and Health Surveys (DHS) database, a global database supported by the international DHS project for collecting, organizing, and disseminating demographic and health-related survey data. Comprising data from over 50 countries, this database includes a wealth of population and health survey information spanning both developing and developed countries worldwide. The relevant data on women’s health in PNG used in this study were extracted from the DHS database, specifically the 2016–2018 Papua New Guinea Demographic and Health Survey (PNGDHS) data. The PNGDHS dataset includes a total of 16,021 households surveyed, yielding 22,531 sample data points. For the purposes of the study, we excluded men, women who are not part of the age group 15–49 years old and who have not given birth to at least one child in the past five years, as well as missing values [13]. Ultimately, A total of 5248 reproductive-age women who had at least one birth in the 5 years preceding the country survey were considered the analytical sample. Details of the methodology, pretesting, training of field workers, sampling design and selection are available in the PNGDHS final report [14].

### Variables

#### Outcome variables

Three outcome variables were considered in this study: whether the women used ANC, SBA and PNC services for the last birth in 5 years preceding the survey. All the outcome variables were binary (Table 1).

**Table 1:** Variable categorisation and levelling

Variables	Categories
**Outcome variable**	
ANC, SBA and PNC services utilisation	ANC: receiving at least four or more ANC visits Categorised into two levels: No, Yes
	SBA: delivery assisted by a skilled health personnel Categorised into two levels: No, Yes
	PNC: having a postnatal check-up after delivery Categorised into two levels: No, Yes
**Explanatory variables**	
**Predisposing factors**	
Age	Categorised into three levels: 15–19 years, 20–34 years, 35–49 years
Marital status	Categorised into two levels: Cohabiting, Married
Working	Categorised into two levels: Not working, Working
Education	Categorised into four levels: No education, Primary, Secondary, Higher
Religion	Categorised into three levels: No religion, Christianity, Non-Christianity
** Enabling factors**	
Media exposure	Categorised into two levels: No, Yes
Getting money needed for treatment	Categorised into two levels: Big problem, Not a big problem
Distance to health facility	Categorised into two levels: Big problem, Not a big problem
Decision maker on healthcare	Categorised into two levels: Not alone, Alone
Household wealth	Categorised into five levels: Poorest, Poorer, Middle, Richer, Richest
Health insurance	Categorised into two levels: No, Yes
Region	Categorised into four levels: Southern region, Highlands region, Momase region, Islands region
Residence	Categorised into two levels: Rural, Urban
** Need factors**	
Parity	Categorised into three levels: 1, 2, ≥ 3
Pregnancy terminations	Categorised into two levels: No, Yes

#### Explanatory variables

On the basis of the conclusions of previous relevant studies [8, 9, 15] and availability in the PNGDHS, 15 explanatory variables were considered and classified into three categories according to Andersen’s behavioural model in this study (Table 1).

### Statistical analyses

Data cleaning, management and analysis were conducted with SPSS version 26.0. Descriptive statistics using frequencies and percentages were presented for the background characteristics and the prevalence of ANC, SBA and PNC services. The association between explanatory and outcome variables was examined by applying the Chi-square test. Variables that showed a statistically significant association (*p* < 0.05) were considered for multivariable logistic regression analysis. The multivariable logistic regression analysis results were presented using adjusted odds ratios (AORs) with 95% confidence interval (95% CI). Using the variance inflation factor (VIF), the multicollinearity test showed no evidence of collinearity amongst the explanatory variables (mean VIF = 1.30, maximum VIF = 1.77, and minimum VIF = 1.01). Dominance analysis was conducted to determine the relative contribution of predisposing, enabling and need factors in multivariable logistic regression. The analysis can decompose each variable accounting for the total variance into the percentage of explained variance by calculating and comparing the average additional contribution (△*R*^2^) of variables relative to all possible subset models [16]. The sample weight was used to account for the complex survey design and generalisability of the findings.

## Results

### Socio-demographic characteristics of the study participants

Table 2 presents the socio-demographic characteristics of the study participants. The results indicated that 69.2% of the women were aged 20–34, and 82.8% were married. Approximately 66.4% were not working, and 49.5% had a primary level of education. The majority (99.0%) of the women were Christians.

**Table 2:** Socio-demographic characteristics and prevalence of MHS amongst women in Papua New Guinea

Variable	Frequency (N)	Percentage(%)	ANC (52.3%)			SBA (58.7%)			PNC (26.6%)		
			Prevalence(%)	χ^2^ -value	p-value	Prevalence(%)	χ^2^ -value	*p*-value	Prevalence(%)	χ^2^ -value	*p*-value
**Predisposing factors**											
Age				3.482	0.175		16.396	< 0.001		2.776	0.250
15–19	176	3.4	55.2			60.9			31.3		
20–34	3634	69.2	53.0			60.3			26.1		
35–49	1438	27.4	50.4			54.2			27.1		
Marital status				0.270	0.603		0.900	0.343		3.411	0.065
Cohabiting	901	17.2	51.6			57.3			24.2		
Married	4347	82.8	52.5			59.0			27.1		
Working				57.884	< 0.001		60.358	< 0.001		4.502	0.034
Not working	3483	66.4	48.9			55.2			25.7		
Working	1765	33.6	60.0			66.4			28.5		
Education				427.682	< 0.001		870.936	< 0.001		93.464	< 0.001
No education	1105	21.1	31.1			31.8			18.2		
Primary	2600	49.5	54.3			58.1			26.9		
Secondary	1330	25.3	70.8			84.4			33.9		
Higher	213	4.1	65.5			98.2			36.7		
Religion				16.220	< 0.001		13.690	0.001		13.695	0.001
No religion	23	0.4	21.9			36.4			6.3		
Christianity	5198	99.0	52.6			58.9			26.8		
Non-Christianity	27	0.5	34.4			36.4			6.3		
**Enabling factors**											
Media exposure				306.763	< 0.001		561.678	< 0.001		90.563	< 0.001
No	2622	50.0	41.1			43.7			21.2		
Yes	2626	50.0	65.0			75.5			32.6		
Getting money needed for treatment				175.830	< 0.001		353.069	< 0.001		24.360	< 0.001
Big problem	3222	61.4	45.5			49.1			24.3		
Not a big problem	2026	38.6	64.3			75.4			30.5		
Distance to health facility				268.352	< 0.001		518.211	< 0.001		50.176	< 0.001
Big problem	2845	54.2	43.1			45.9			23.0		
Not a big problem	2403	45.8	65.8			77.0			31.7		
Decision maker on healthcare				18.089	< 0.001		22.133	< 0.001		0.056	0.812
Not alone	3787	72.2	50.5			56.6			26.7		
Alone	1461	27.8	56.8			63.6			26.3		
Household wealth				337.467	< 0.001		1051.716	< 0.001		100.434	< 0.001
Poorest	875	16.7	32.5			32.0			17.9		
Poorer	884	16.8	47.0			40.9			22.1		
Middle	993	18.9	54.5			58.1			28.7		
Richer	1249	23.8	60.3			72.5			29.6		
Richest	1247	23.8	69.3			92.8			35.1		
Health insurance				41.417	< 0.001		84.370	< 0.001		5.555	0.018
No	5055	96.3	51.6			57.6			26.3		
Yes	193	3.7	77.0			93.3			34.5		
Region				197.642	< 0.001		228.819	< 0.001		82.969	< 0.001
Southern region	1518	28.9	62.4			67.8			32.9		
Highlands region	1375	26.2	48.8			55.8			20.4		
Momase region	1153	22.0	42.1			47.3			27.0		
Islands region	1202	22.9	68.9			77.2			34.1		
Residence				66.628	< 0.001		224.019	< 0.001		45.757	< 0.001
Rural	4060	77.4	50.4			55.2			25.1		
Urban	1188	22.6	68.3			87.5			38.3		
**Need factors**											
Parity				61.841	< 0.001		166.144	< 0.001		7.092	0.029
1	1071	20.4	60.9			70.7			29.6		
2	1103	21.0	55.7			66.9			25.5		
≥ 3	3074	58.6	48.1			51.5			25.8		
Pregnancy terminations				9.694	0.002		14.445	< 0.001		3.977	0.046
No	4965	94.6	51.8			58.1			26.3		
Yes	283	5.4	61.3			69.5			31.7		

Source: 2016–2018 Papua New Guinea Demographic and Health Survey

### Prevalence of MHS amongst women in PNG

Table 2 also presents the prevalence of MHS amongst women in PNG. The prevalence rates of ANC, SBA and PNC services were 52.3%, 58.7% and 26.6%, respectively. At the bivariate level, all the explanatory variables apart from age and marital status showed statistically significant associations (*p* < 0.05) with ANC services utilisation. All the explanatory variables apart from marital status showed statistically significant associations (*p* < 0.05) with SBA services utilisation. All the explanatory variables apart from age, marital status and decision maker on healthcare showed statistically significant associations (*p* < 0.05) with PNC services utilisation.

### Determinants of MHS utilisation in PNG

Table 3 presents the multivariable analysis of the determinants of MHS utilisation in PNG. For ANC services, working women (AOR = 1.268, CI = 1.112–1.445) and those with a secondary level of education (AOR = 2.370, CI = 1.933–2.905) had higher odds of services utilisation than their counterparts. Women exposed to media (AOR = 1.473, CI = 1.280–1.695), those who did not have a big problem getting money needed for treatment (AOR = 1.169, CI = 1.005–1.359), those who did not have a big problem in terms of distance to health facility (AOR = 1.580, CI = 1.361–1.835), those who made healthcare decisions alone (AOR = 1.160, CI = 1.020–1.320), those in the richest wealth quintile (AOR = 1.455, CI = 1.139–1.859), those who had health insurance (AOR = 1.615, CI = 1.071–2.435) and those from Islands region (AOR = 1.254, CI = 1.012–1.555) were more likely to use the services than their counterparts. On the contrary, women from the Highlands region (AOR = 0.782, CI = 0.660–0.926) and Momase region (AOR = 0.514, CI = 0.432–0.611) and those with a parity of 2 (AOR = 0.803, CI = 0.668–0.964) and ≥ 3 (AOR = 0.677, CI = 0.582–0.788) were less likely to use the services than their counterparts.

**Table 3:** Multivariable analysis of the determinants of MHS utilisation in Papua New Guinea

Variable	ANC	SBA	PNC
	AOR(95% CI)	AOR(95% CI)	AOR(95% CI)
**Predisposing factors**			
Age			
15–19	-	Ref	-
20–34	-	1.181 (0.833–1.675)	-
35–49	-	1.374 (0.939–2.011)	-
Working			
Not working	Ref	Ref	Ref
Working	1.268*** (1.112–1.445)	1.185* (1.021–1.374)	0.952 (0.829–1.092)
Education			
No education	Ref	Ref	Ref
Primary	1.880*** (1.616–2.186)	2.190*** (1.869–2.567)	1.221* (1.025–1.454)
Secondary	2.370*** (1.933–2.905)	3.964*** (3.142–5.002)	1.351** (1.084–1.683)
Higher	1.211 (0.845–1.737)	12.363*** (4.601–33.224)	1.602* (1.115–2.300)
Religion			
No religion	Ref	Ref	Ref
Christianity	1.824 (0.765–4.354)	0.840 (0.376–1.874)	4.025* (1.020–15.876)
Non-Christianity	1.115 (0.344–3.613)	0.472 (0.144–1.552)	0.918 (0.132–6.405)
**Enabling factors**			
Media exposure			
No	Ref	Ref	Ref
Yes	1.473*** (1.280–1.695)	1.289*** (1.109–1.499)	1.317*** (1.131–1.533)
Getting money needed for treatment			
Big problem	Ref	Ref	Ref
Not a big problem	1.169* (1.005–1.359)	1.089 (0.918–1.292)	0.920 (0.786–1.076)
Distance to health facility			
Big problem	Ref	Ref	Ref
Not a big problem	1.580*** (1.361–1.835)	1.655*** (1.402–1.954)	1.242** (1.060–1.455)
Decision maker on healthcare			
Not alone	Ref	Ref	
Alone	1.160* (1.020–1.320)	1.091 (0.944–1.262)	-
Household wealth			
Poorest	Ref	Ref	Ref
Poorer	1.429*** (1.190–1.716)	1.089 (0.902–1.315)	1.135 (0.916–1.407)
Middle	1.542*** (1.277–1.860)	1.848*** (1.525–2.240)	1.444*** (1.167–1.787)
Richer	1.375** (1.120–1.689)	2.390*** (1.925–2.969)	1.212 (0.962–1.526)
Richest	1.455**(1.139–1.859)	6.947*** (5.066–9.527)	1.283 (0.988–1.667)
Health insurance			
No	Ref	Ref	Ref
Yes	1.615* (1.071–2.435)	1.506 (0.736–3.082)	0.908 (0.633–1.304)
Region			
Southern region	Ref	Ref	Ref
Highlands region	0.782** (0.660–0.926)	1.036 (0.856–1.253)	0.602*** (0.504–0.720)
Momase region	0.514*** (0.432–0.611)	0.505*** (0.415–0.614)	0.821* (0.687–0.981)
Islands region	1.254* (1.012–1.555)	1.414** (1.106–1.808)	1.046 (0.849–1.289)
Residence			
Rural	Ref	Ref	Ref
Urban	1.120 (0.898–1.396)	1.650** (1.214–2.242)	1.249* (1.011–1.543)
**Need factors**			
Parity			
1	Ref	Ref	Ref
2	0.803* (0.668–0.964)	0.819 (0.661–1.015)	0.818* (0.675–0.992)
≥ 3	0.677*** (0.582–0.788)	0.494*** (0.408–0.597)	0.911 (0.779–1.066)
Pregnancy terminations			
No	Ref	Ref	Ref
Yes	1.193 (0.912–1.560)	1.134 (0.829–1.551)	1.135 (0.868–1.485)

*AOR* Adjusted odds ratio, 95% confidence intervals in brackets, *Ref* Reference

^*^p < 0.05, **p < 0.01, ***p < 0.001

Source: 2016–2018 Papua New Guinea Demographic and Health Survey

For SBA services, working women (AOR = 1.185, CI = 1.021–1.374) and those with a higher level of education (AOR = 12.363, CI = 4.601–33.224) had higher odds of services utilisation than their counterparts. Similarly, women who were exposed to media (AOR = 1.289, CI = 1.109–1.499), those who did not have a big problem in terms of distance to health facility (AOR = 1.655, CI = 1.402–1.954), those in the richest wealth quintile (AOR = 6.947, CI = 5.066–9.527), those from the Islands region (AOR = 1.414, CI = 1.106–1.808) and urban women (AOR = 1.650, CI = 1.214–2.242) were more likely to use the services than their counterparts. However, women from the Momase region (AOR = 0.505, CI = 0.415–0.614) and those with a parity of ≥ 3 (AOR = 0.494, CI = 0.408–0.597) were less likely to use the services than their counterparts.

For PNC services, women with a higher level of education (AOR = 1.602, CI = 1.115–2.300) and those who believe in Christianity (AOR = 4.025, CI = 1.020–15.876) had higher odds of services utilisation than their counterparts. Women who were exposed to media (AOR = 1.317, CI = 1.131–1.533), those who did not have a big problem in terms of distance to health facility (AOR = 1.242, CI = 1.060–1.455), those in the middle wealth quintile (AOR = 1.444, CI = 1.167–1.787) and urban women (AOR = 1.249, CI = 1.011–1.543) were more likely to use the services than their counterparts. On the contrary, women from the Highlands region (AOR = 0.602, CI = 0.504–0.720) and Momase region (AOR = 0.821, CI = 0.687–0.981) and those with a parity of 2 (AOR = 0.818, CI = 0.675–0.992) were less likely to use the services than their counterparts.

Table 4 presents the dominance analysis of the relative contribution of predisposing, enabling and need factors associated with MHS utilisation in PNG. According to the percentage of explained variance, ANC, SBA and PNC services utilisation were all primarily influenced by enabling factors, followed by predisposing and need factors.

**Table 4:** Dominance analysis of the relative contribution of predisposing, enabling and need factors associated with MHS utilisation in Papua New Guinea

Subset Model	ANC				SBA				PNC			
		△$\boldsymbol R_{\mathbf N}^{\mathbf2}$				△$\boldsymbol R_{\mathbf N}^{\mathbf2}$				△$\boldsymbol R_{\mathbf N}^{\mathbf2}$		
	$\mathbf R_{\mathbf N}^{\mathbf2}$	X_1_	X_2_	X_3_	$\textbf{R}_{\mathbf N}^{\mathbf2}$	X_1_	X_2_	X_3_	$\textbf{R}_{\mathbf N}^{\mathbf2}$	X_1_	X_2_	X_3_
Null and k = 0 average	0	0.1114	0.1579	0.0186	0	0.2245	0.3272	0.0471	0	0.0291	0.0520	0.0031
X_1_	0.1114	-	0.0722	0.0072	0.2245	-	0.1167	0.0163	0.0291	-	0.0292	0.0012
X_2_	0.1579	0.0257	-	0.0071	0.3272	0.0140	-	0.0168	0.0520	0.0063	-	0.0016
X_3_	0.0186	0.1000	0.1464	-	0.0471	0.1937	0.2969	-	0.0031	0.0272	0.0505	-
k = l average	-	0.0629	0.1093	0.0072	-	0.1039	0.2068	0.0166	-	0.0168	0.0399	0.0014
X_1_X_2_	0.1836	-	-	0.0058	0.3412	-	-	0.0369	0.0583	-	-	0.0013
X_1_X_3_	0.1186	-	0.0708	-	0.2408	-	0.1373	-	0.0303	-	0.0293	-
X_2_X_3_	0.1650	0.0244	-	-	0.3440	0.0341	-	-	0.0536	0.0060	-	-
k = 2 average	-	0.0244	0.0708	0.0058	-	0.0341	0.1373	0.0369	-	0.0060	0.0293	0.0013
X_1_X_2_X_3_	0.1894	-	-	-	0.3781	-	-	-	0.0596	-	-	-
Overall average	-	0.0662	0.1127	0.0105	-	0.1208	0.2238	0.0335	-	0.0173	0.0404	0.0019
Percentage of explained variance (%)	-	34.95	59.50	5.54	-	31.95	59.19	8.86	-	29.03	67.79	3.19

*X*_*1*_ Predisposing factors, *X*_*2*_ Enabling factors, *X*_*3*_ Need factors, ${R}_{N}^{2}$, Nagelkerke R^2^

## Discussion

The study sought to assess the prevalence and determinants of ANC, SBA and PNC services amongst women in PNG. Unlike other similar studies [8, 9], we carried out dominance analysis to determine the relative contribution of predisposing, enabling and need factors in multivariable logistic regression. The results could provide new evidence to inform the development of targeted policies and interventions. The study results revealed that the prevalence rate of ANC services amongst women in PNG was 52.3%. A similar indicator value was reported by the World Health Organization (WHO), where 49.0% of reproductive-age women had at least four or more ANC visits in PNG (2011–2018) [17]. The prevalence rate of SBA services (58.7%) in this study is higher than in two studies, with an estimate of 28% (2007–2010) [18] and 39.1% (2009–2012) [19] in PNG. The differences in the study findings could be explained by the differences in the timing of studies. Increased SBA services utilisation might be due to improved health human resources. Since 2009, the quality of midwifery training in PNG has improved dramatically, and the number of practising midwives has almost tripled, helping to make inroads on addressing the serious staff shortage [20]. PNC services are one important measure of access to care but have received very little attention in PNG in formal research. This study’s prevalence rate of PNC services was 26.6%, much lower than the sub-Saharan Africa average rate (52.5%) [21]. In addition, we found that MHS utilisation was more significantly explained by enabling factors than predisposing and need factors.

The study found that levels of education were significantly associated with ANC, SBA and PNC services utilisation. Specifically, women with higher levels of education showed a higher probability of using MHS than those without education. This finding was consistent with other similar studies in LMICs such as Cambodia [22], Ethiopia [23] and sub-Saharan Africa [24]. Education makes women more aware of the importance of MHS and receptive to health promotion concepts [25]. Hence, educated women have the ability to identify disease symptoms, take preventive measures and seek appropriate healthcare [26].

Media exposure was found to influence the likelihood of all three MHS services utilisation. Women exposed to mass media such as newspapers, magazines, radio or television had higher odds of MHS compared with those who did not have access to the media. Mass media can disseminate health information to the public and create a family and social environment conducive to health [27]. Some previous studies suggested that women exposed to the mass media can obtain more health knowledge, information and family support than those who were unexposed [28, 29].

The study showed that the utilisation of ANC, SBA and PNC services was influenced by distance to the health facility. Women who indicated that distance was not a big problem in terms of healthcare were more likely to use MHS than their counterparts. Amongst the determinants of not utilizing appropriate maternal healthcare services in Sub-Saharan Africa, geographical accessibility to health facilities is also considered a major obstacle [30, 31]. In PNG, the lack of transport and health infrastructure increased the distance to healthcare facilities, leading to lowered motivation to seek MHS amongst women.

Women’s household wealth was positively associated with all three MHS services utilisation. Specifically, women with a higher wealth status had a higher likelihood of ANC, SBA and PNC services usage than women with a lower wealth status. This finding was consistent with previous studies in other countries [32, 33]. Despite the availability of free primary healthcare in PNG since 2014 [34], women from lower wealth households still face financial barriers to using maternal health services, such as transportation and other related costs. On the other hand, wealthier women will certainly not face these barriers, as they have an increased ability to afford the costs associated with healthcare.

Region and residence were also highly significant indicators of MHS utilisation. Compared with women from the Southern region, women from the Islands region were more likely to use MHS, whereas women from Highlands region and Momase region were less likely to use MHS. Rural women had lower odds of SBA and PNC services than urban women. Similar findings were reported in previous studies conducted in Nepal [35] and Ethiopia [36]. This finding could be attributed to the unbalanced development of the areas in PNG [13]. Most provinces in Highlands region and Momase region are relatively economically backward and geographically remote, and women’s willingness to use MHS was relatively low. Similarly, the majority of PNG’s population (80%) live in rural areas with inadequate healthcare facilities making the provision of healthcare additionally challenging [37].

The study also found that the utilisation of ANC, SBA and PNC services decreased with increasing parity. Some researchers pointed out that women who do not experience any complications for a previous pregnancy might not seek MHS during their current pregnancy [38]. The other reason could be that having more children leads to resource constraints [39], which has a negative effect on MHS utilisation. In addition, employment strongly predicted MHS utilisation. Similar to previous studies [34, 40], working women increased the chances of ANC and SBA services usage.

### Strengths and limitations

The study used nationally representative data so the findings can be generalised to all reproductive-age women in PNG. Guided by Andersen’s behaviour model, the study conducted dominance analysis to determine the relative contribution of predisposing, enabling and need factors associated with MHS utilisation. Despite these strengths, the study is cross-sectional, and causal relationships cannot be inferred. Given the use of secondary data, we did not consider more relevant variables associated with MHS utilisation, which might reduce the relative contribution of need factors.

## Conclusions

This study demonstrated that enabling factors such as media exposure, distance to health facility, household wealth, region and residence have the greatest impact on MHS utilisation, followed by predisposing (working, education) and need factors (parity). Therefore, enabling factors should be prioritised when developing maternal health programmes and policies. For example, transport and health infrastructure should be strengthened and women’s education and vocational training should be increased, especially in Highlands region, Momase region and rural areas, to increase the utilisation of MHS.

## Data Availability

The datasets generated and analyzed during the current study are available in the Demographic and Health Survey programme repository, https://www.dhsprogram.com/methodology/survey/survey-display-499.cfm.

## Abbreviations

SDGs: Sustainable Development Goals
LMICs: Low- and Middle-Income Countries
PNG: Papua New Guinea
ANC: Antenatal Care
SBA: Skilled Birth Attendant
PNC: Postnatal Care
MHS: Maternal Healthcare Services
PNGDHS: Papua New Guinea Demographic and Health Survey
AORs: Adjusted Odds Ratios
95% CI: 95% Confidence Interval
VIF: Variance Inflation Factor
WHO: World Health Organization
